# Epileptic Seizures As the Sole Presentation of a Radiologically Isolated Syndrome: A Case Report and Review of the Literature

**DOI:** 10.7759/cureus.75226

**Published:** 2024-12-06

**Authors:** Asmaa Hazim, Yasmine Mimouni, Meriem Hakimi, Sarra Saaf, Meryem El Azhari, Zineb El Yakoubi, Sara Lhassani, Loubna Kazzoul, Jehanne Aasfara, Hamid Ouhabi

**Affiliations:** 1 Department of Neurology, Mohamed VI University of Health Sciences (UM6SS), Casablanca, MAR; 2 Department of Neurology, Cheikh Khalifa Bin Zayed Hospital, Casablanca, MAR; 3 Department of Neurology, Cheikh Khalifa International University Hospital, Casablanca, MAR; 4 Department of Neurology, Cheikh Khalifa Ibn Zayed Hospital, Faculty of Medicine, Mohamed VI University of Health Sciences, Casablanca, MAR; 5 Department of Neurology, Cheikh Khalifa International University Hospital, Mohamed VI University of Health Sciences (UM6SS), Casablanca, MAR; 6 Department of Neurology, Cheikh Khalifa International University Hospital, Faculty of Medicine, Mohamed VI University of Health Sciences (UM6SS), Casablanca, MAR

**Keywords:** antiepileptic drugs (aed), epilepsy, multiple sclerosis, new-onset seizure, radiologically isolated syndrome

## Abstract

Multiple sclerosis (MS) is the most prevalent long-term inflammatory condition affecting the central nervous system in adults. However, seizures are rarely described as the first presentation of MS or as a sole manifestation of radiologically isolated syndrome (RIS) or clinically isolated syndrome (CIS). The diagnosis of MS typically requires clinical evidence of neurological deficits and supportive radiological findings; however, RIS is characterized by incidental magnetic resonance imaging (MRI) findings suggestive of MS in the absence of clinical symptoms.

The management of RIS remains a subject of ongoing debate. Although the majority of individuals with RIS remain clinically asymptomatic, the presence of radiological lesions suggests a potential risk for progression to clinically definite MS. The decision to initiate disease-modifying therapies (DMTs) in RIS is influenced by factors such as lesion burden, lesion characteristics, and patient risk factors for conversion to MS.

The association between RIS and epilepsy is not well established, and the timing of initiating long-term treatment in such cases remains uncertain. In cases where seizures occur in the context of RIS or CIS, it is important to balance the treatment of epilepsy with the careful monitoring of disease progression. While antiepileptic drugs (AEDs) may be necessary to control seizures, early initiation of DMTs may be considered to prevent further neurological damage and clinical exacerbations, particularly in patients with high-risk features on MRI.

We report the case of a 29-year-old woman with no previous medical history who presented with an inaugural generalized tonic-clonic seizure with numerous MS-like demyelinating lesions in the supratentorial, brainstem and medullary areas and the presence of cerebrospinal fluid-specific oligoclonal bands. The AEDs were started after the second occurrence of seizures, raising the question of the mean time to start long-term treatment in MS/RIS/CIS disease.

## Introduction

The course of multiple sclerosis (MS) is highly variable, with the possibility of numerous comorbidities along the way [1]. The relationship between epilepsy and MS is well established. Research has expanded since its first description 30 years ago, strengthening the link between these two entities [2].

Radiologically isolated syndrome (RIS) refers to the incidental detection of demyelinating lesions on magnetic resonance imaging (MRI) suggestive of MS in individuals without clinical symptoms. Initially described in the early 2000s, RIS has since emerged as a distinct radiological entity with potential implications for predicting the future development of MS. Although the neurological manifestations of MS are well documented, the role of RIS in predisposing individuals to other neurological conditions, such as epilepsy, remains underexplored.

Recent advances in neuroimaging have provided deeper insights into the structural and functional disruptions underlying RIS and epilepsy. Shared pathophysiological mechanisms, such as cortical demyelination, inflammation, and neuronal hyperexcitability, may bridge these two conditions. However, the relationship between RIS and epilepsy remains poorly understood, warranting further investigation to clarify potential clinical implications and improve patient management strategies.

In the context of MS, cumulative incidence increases with disease duration up to nearly 6% [3], although a few rare cases of convulsive seizures have been reported as early manifestations of the disease, especially in the infantile form of MS [4], even if they are not classically considered as a clinical attack of the disease.

## Case presentation

We present the case of a 29-year-old female schoolteacher with no significant medical history, no indications of systemic disease, and no known risk factors for epilepsy. She experienced a nocturnal unprovoked generalized tonic-clonic seizure, preceded by a scream-like vocalization, lasting approximately three minutes and followed by a period of postictal confusion. The postictal neurological examination was normal, and the rest of the examination did not show clinical signs indicative of a systemic disease. The EEG performed on the day of the seizure was normal.

**Figure 1:**
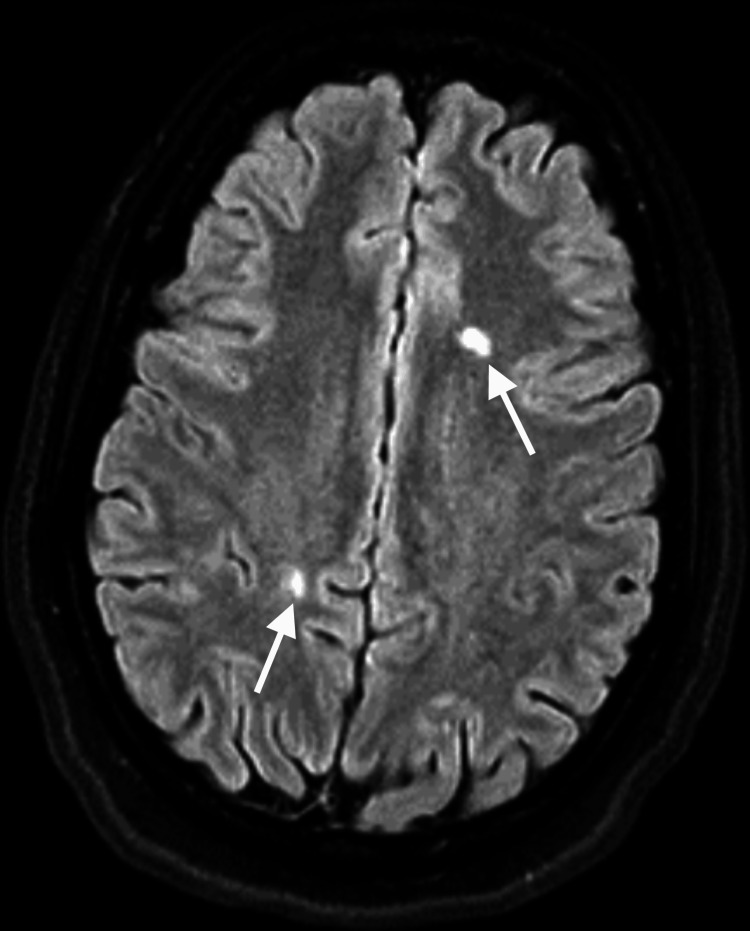
Postictal brain MRI (axial section, FLAIR) Two roughly oval-shaped lesions, in hypersignal FLAIR, with their long axis perpendicular to the ventricular axis, affecting the centrum semiovale MRI: magnetic resonance imaging; FLAIR: fluid-attenuated inversion recovery

**Figure 2:**
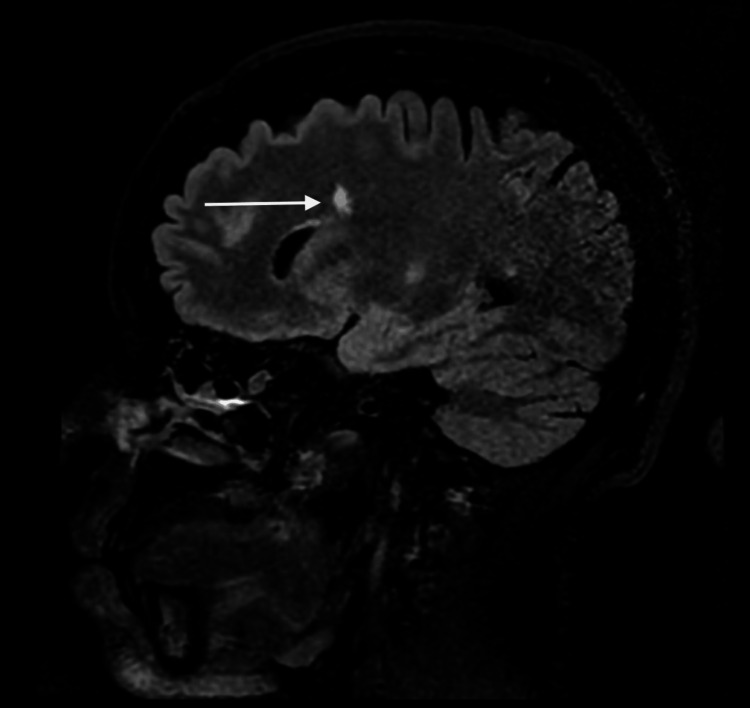
Brain MRI performed after the first seizure (sagittal section, FLAIR) Left paraventricular FLAIR intensity, with a long axis perpendicular to the ventricular axis (white arrow) MRI: magnetic resonance imaging; FLAIR: fluid-attenuated inversion recovery

**Figure 3:**
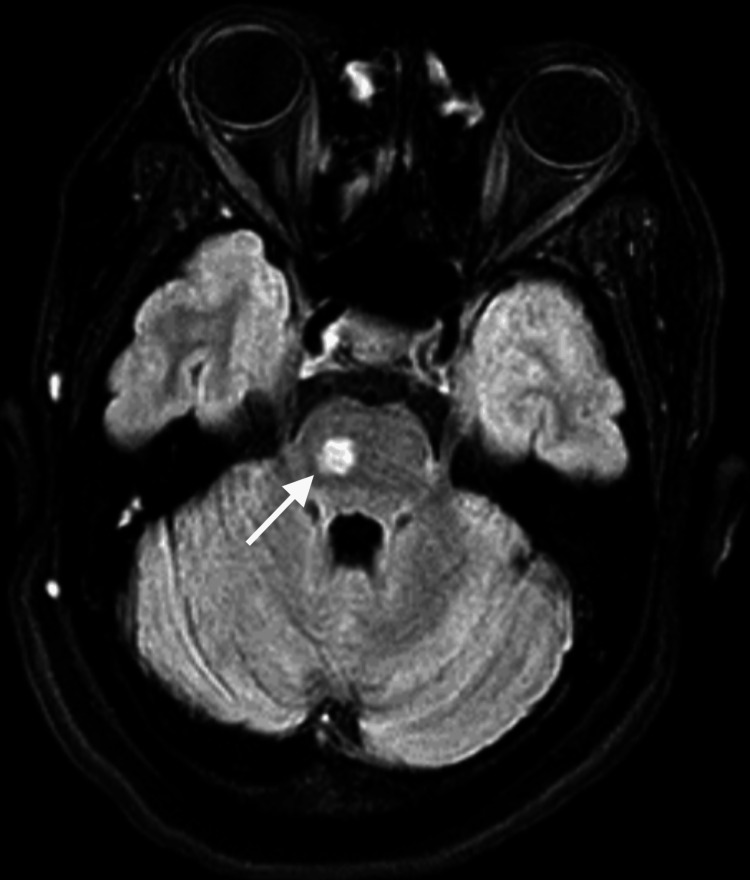
Brain MRI performed after the first seizure (axial section, FLAIR) Right lateral pontine lesion with FLAIR hyperintensity (white arrow) MRI: magnetic resonance imaging; FLAIR: fluid-attenuated inversion recovery

**Figure 4:**
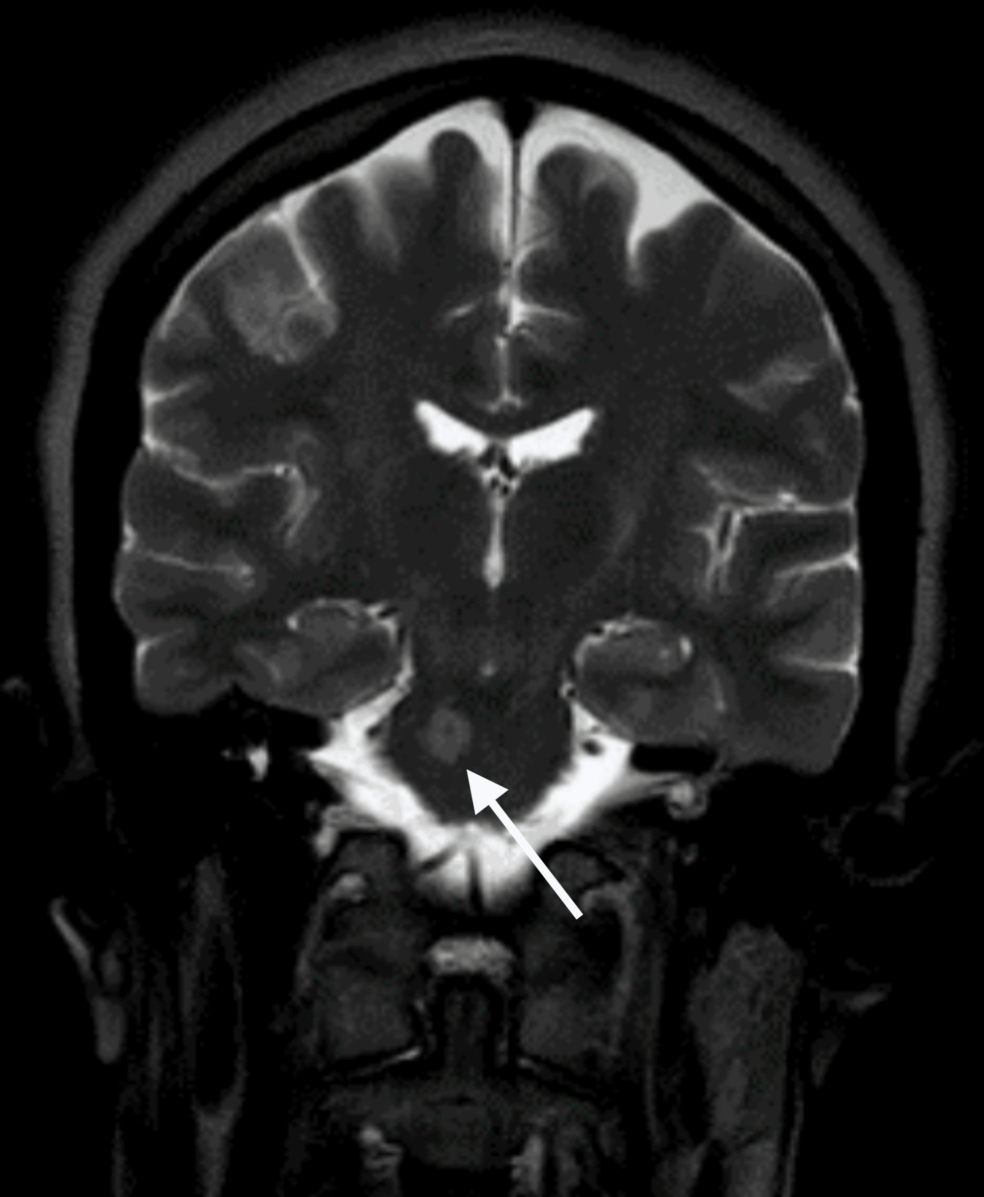
Brain MRI performed postictal (coronal section, T2 imaging) The same right lateral pontine lesion, as seen in Figure 3, with T2 hyperintensity (white arrow) MRI: magnetic resonance imaging

**Figure 5:**
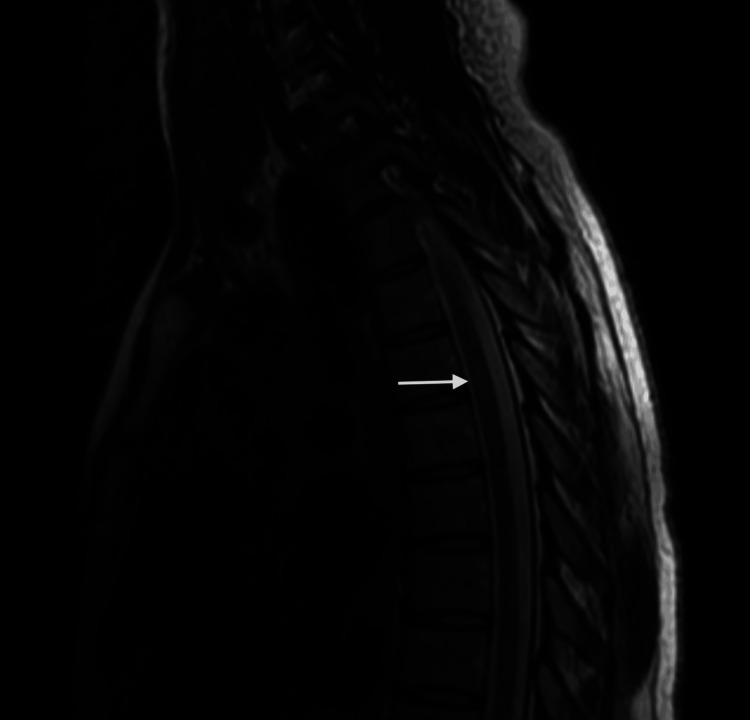
Postictal dorsal spine magnetic resonance (sagittal section, T2 imaging) Slight T2 hyperintensity within the spinal cord at the level of the T5-T6 disc, extending over less than one vertebral body (white arrow)

**Figure 6:**
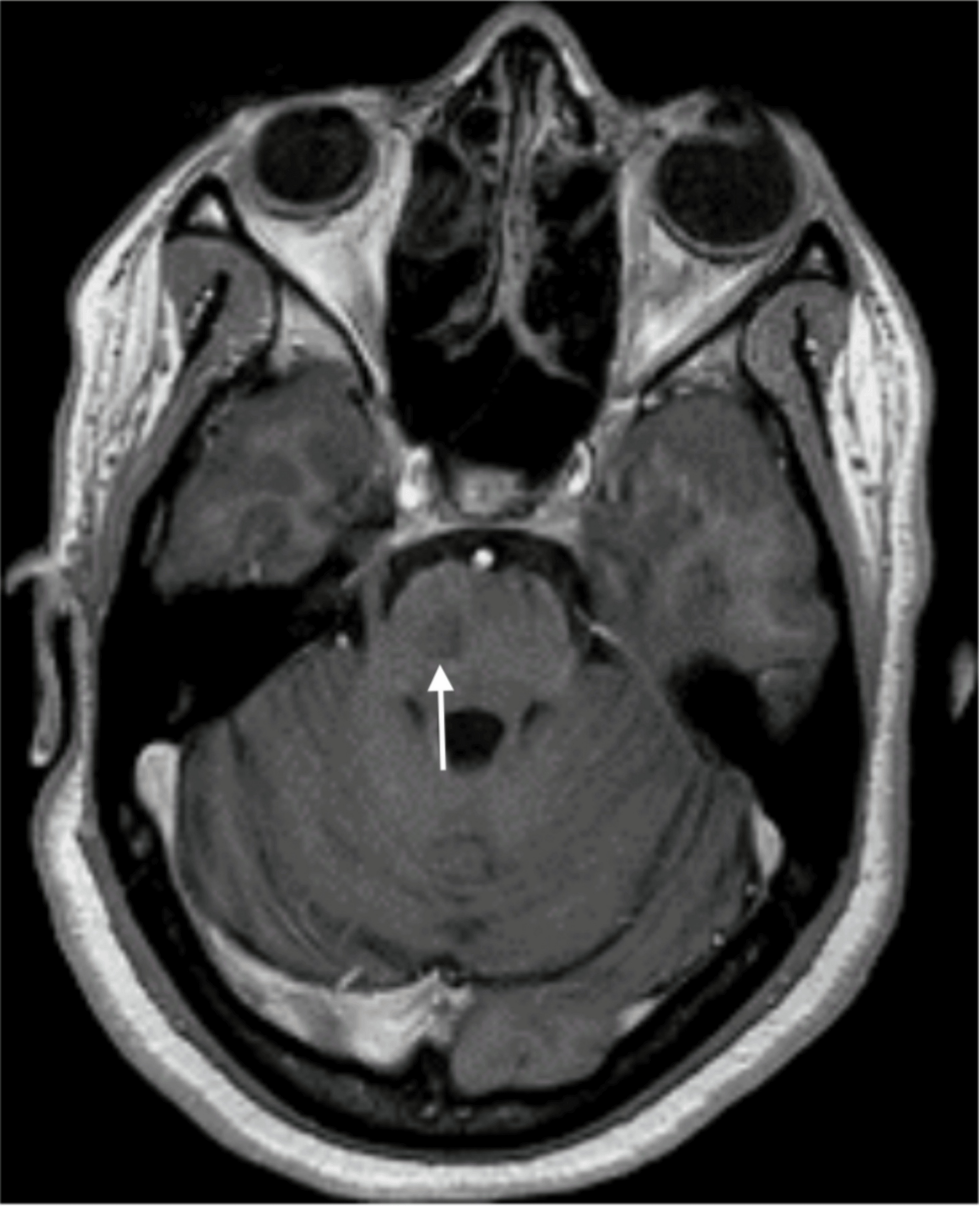
Postictal brain MRI centered on the pons (axial section, T1-weighted enhanced imaging) The same right lateral pontine lesion, as seen in Figures 3, 4, shows no enhancement post-gadolinium injection (white arrow) MRI: magnetic resonance imaging

**Figure 7:**
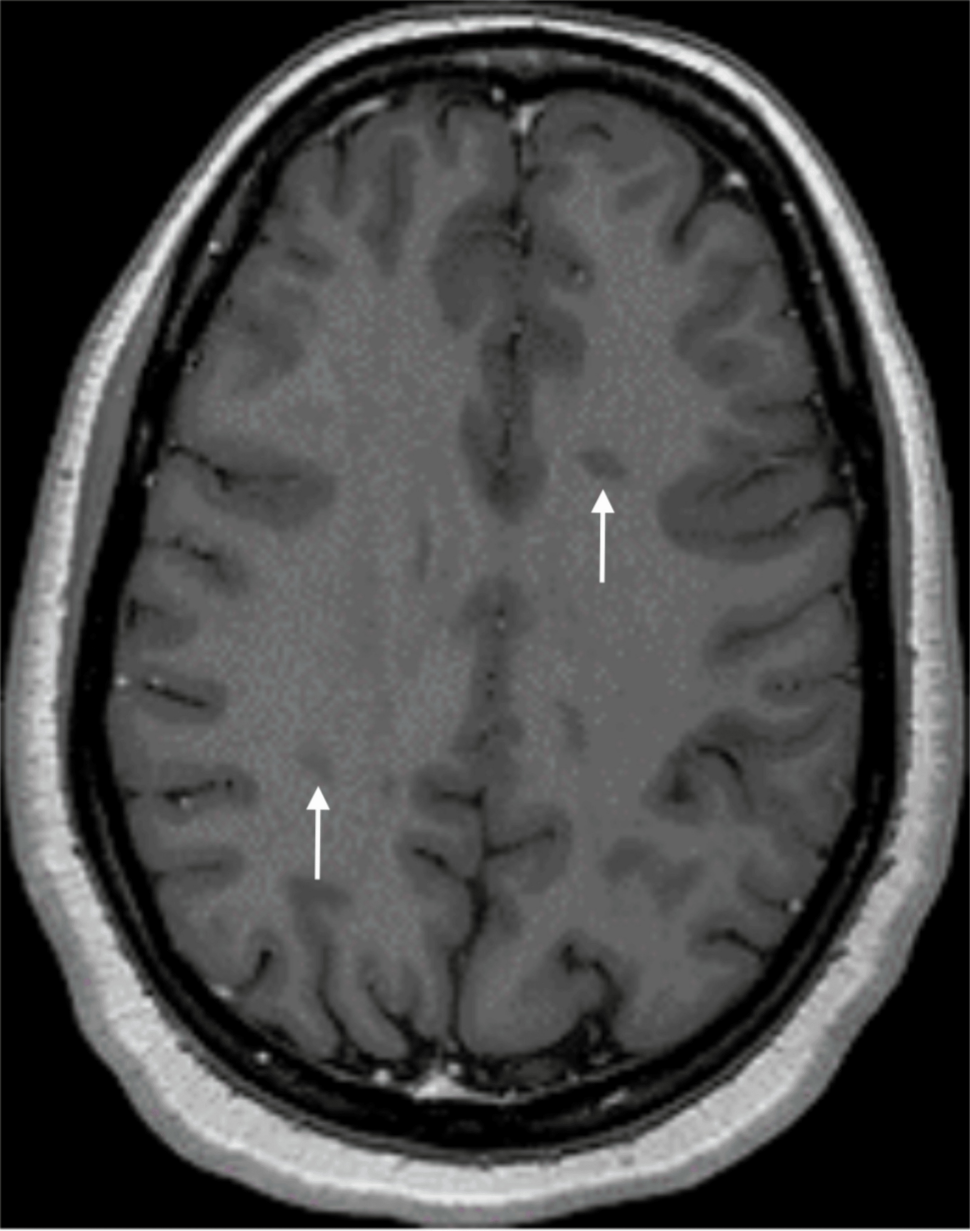
Postictal brain MRI (axial section, T1-weighted enhanced imaging) Bilateral centrum semiovale lesions, as seen in Figure 1, show no postcontrast enhancement (white arrows) MRI: magnetic resonance imaging

Brain and spine MRI conducted after this episode using a 1.5-Tesla MRI revealed multiple well-defined, ovoid, periventricular, and subcortical demyelinating lesions in the white matter with a hyperintense signal on fluid-attenuated inversion recovery (FLAIR) sequence (Figures 1, 2), including a lateralized right pontine lesion (Figures 3, 4) and a dorsal spinal cord lesion (Figure 5), without any enhancement (Figures 6, 7) and without corresponding signals on other sequences. These lesions met the criteria for dissemination in space (DIS) as defined by the 2024 revised McDonald criteria. However, given the occurrence of a single clinical episode and the availability of only one brain MRI, the criteria for dissemination in time were not met. Nevertheless, this did not preclude the diagnosis, as temporal dissemination is no longer required to confirm MS under the revised criteria.

The preliminary results of the laboratory tests were unremarkable. Infectious and immunological blood workups were negative. However, cerebrospinal fluid (CSF) analysis revealed the presence of oligoclonal IgG bands.

The diagnosis of RIS was established according to the revised 2023 RIS criteria [5], with a high risk of progression to confirmed MS (age <39 years, presence of spinal cord and brainstem lesions, and detection of oligoclonal bands in CSF). Consequently, the decision was made to initiate the patient on disease-modifying therapy (DMT; fingolimod 0.5 mg, one capsule per day).

Regarding epilepsy, the patient did not initially experience additional seizures, thus not meeting the criteria for epilepsy according to the International League Against Epilepsy. Therefore, she was not prescribed antiseizure medication. Nevertheless, two months later, she had a second seizure with the same semiological characteristics as the first episode: a nocturnal generalized tonic-clonic seizure preceded by a scream, lasting approximately five minutes. Consequently, she was started on antiseizure drugs, which she is still on (levetiracetam 500 mg per day), and a follow-up brain MRI is planned after three months.

## Discussion

The relationship between MS and epilepsy has been well established over the years through numerous studies [1], while the connection between RIS and epilepsy remains less clear, with several hypotheses proposed to explain this potential association.

The direct involvement of plaques, as well as the role of edema and inflammatory and glial reaction around white matter demyelinated lesions, is supported by both neuroimaging and pathological studies, including a cascade of immune-mediated events, where T-cells, B-cells, and microglia contribute to inflammation and damage to the myelin. The inflammatory response triggers the activation of various glial cells, including astrocytes and oligodendrocytes, which attempt to repair the damaged myelin but often result in glial scar formation, leading to long-term neurological deficits [6]. These studies show that 25% of MS plaques are close to the cerebral cortex, and MRI studies suggested a higher frequency of pure intracortical lesions or lesions extending into the cortex in MS patients with epileptic seizures [7,8]. In the study by Calabrese, intracortical lesions were observed in 90% of patients with relapsing-remitting multiple sclerosis (RRMS) and epilepsy, compared to just 48% of those with RRMS who did not have epilepsy [9].

Unfortunately, cortical lesions are poorly detected in 1.5T MRI, the one used in our facility. The need to use at least a 3T MRI and a double inversion recovery sequence, or, if available, an ultrahigh field imaging (7T or 9.4T) is essential to detect purely cortical lesions that could explain the occurrence of seizures [10].

In parallel with the recognition of gray matter abnormalities, some research has highlighted the impairment of the gamma-aminobutyric acid system in MS (particularly in its econdary progressive multiple sclerosis type), which plays an important role in epilepsy [11]. Other mechanisms that can lead to cellular hyperexcitability, such as dysregulation of the ionic balance or the release of proinflammatory proteins, have also been studied [12].

The risk of seizures increases with the progression of the disease and the number of lesions. Nonetheless, epileptic symptoms can appear even before the onset of MS, which emphasizes the theory that MS can exist long before it first becomes clinically apparent [13].

A few rare reports of inaugural convulsive seizures as the only clinical manifestations for years have been reported in the literature, notably by Al Hussona et al. [14] and Gambardella et al. [15], the latter having described five cases of partial convulsive seizures with intercritical epileptic abnormalities on EEG in all patients.

As for the meantime and disability progression, Grothe et al. have demonstrated that the mean time to the diagnosis of MS was proved lower with seizures onset compared to the other manifestations at onset and that epileptic seizures as the first manifestation of MS are associated with a higher amount of disability progression over time [16].

Furthermore, data in the literature remain poor concerning the association of epileptic manifestations in RIS and/or clinically isolated syndrome (CIS). Al Hussona et al., in a series of four patients, suggested including epileptic seizures as a manifestation of CIS [14].

In our case, the occurrence of an unexplained convulsive seizure associated with the presence of incidental demyelinating lesions in the brain and spinal cord led us to investigate all the differential diagnoses of MS, in particular the neurological manifestations of systemic diseases. Our patient fulfilled Poser’s laboratory-supported definite MS [17] but not the 2017 McDonald revised criteria [18].

The diagnosis of RIS at high MS risk was retained given the presence of white matter demyelinating lesions of morphology and localization characteristic of MS, fulfilling the 2017 McDonald revised criteria of DIS [18]. The demonstration of oligoclonal bands in the CSF enables RIS to be classified at high risk of progression to CIS and/or MS [5].

Some studies suggest initiating long-term antiepileptic therapy after two or three seizures, given that seizures in MS indicate an active demyelinating plaque and considering the favorable prognosis of epilepsy [19].

The occurrence of status epilepticus represents a significant threat to MS patients with epilepsy [19]. It is important to consider that an underlying cortical epileptogenic lesion, which may not be visible on standard brain imaging, could be responsible for both the onset and potential recurrence of seizures. This highlights the need for early intervention in managing seizures, particularly through the initiation of long-term antiepileptic treatment following the first seizure, a practice that contradicts previously established guidelines [7].

In managing epilepsy in MS patients, three antiepileptic drugs are generally preferred: levetiracetam, lamotrigine, and lacosamide [20]. These medications are chosen due to their efficacy and favorable safety profiles in individuals with MS, offering better management of seizures while minimizing potential interactions with DMTs.

While some authors argue that seizures in MS are typically benign and respond well to antiepileptic therapy, Engelsen and Grønning contend that their effectiveness in MS patients is generally poor [13-21].

In our case, our patient has received long-term treatment after the second occurrence of an epileptic seizure, caused probably by a cortical demyelinating lesion, either a new one or an already present one, not visible on the first brain MRI 1.5T performed.

## Conclusions

In conclusion, although seizures have been described during the course of MS, they are not typically considered as initial manifestations of the disease. Our case suggests that epilepsy may present early in the disease course, even in the context of RIS, where patients exhibit demyelinating lesions on imaging but do not display overt clinical symptoms of MS. This highlights the potential for seizures to be an early and possibly isolated manifestation of RIS, necessitating careful consideration in both diagnosis and management.

The literature on the relationship between RIS and epilepsy is currently very limited, with few studies addressing this potential association. Our case adds to the growing body of evidence suggesting that the presence of demyelinating lesions, even in the absence of clinical MS, may predispose patients to epilepsy. This points to the need for further research to better understand the pathophysiological mechanisms linking RIS and epilepsy. Additionally, it is crucial to develop clearer management strategies for patients with RIS who experience seizures, including the timing of long-term antiepileptic treatment and the role of DMTs in preventing further neurological complications.
